# Quinpirole, a D2-like Dopaminergic Receptor Agonist, Regulates Neuroinflammation and Reduces NF-κB Nuclear Expression in Microglia from Hippocampus and Brain Cortex Induced by Rapid Eye Movement Sleep Deprivation in Mice

**DOI:** 10.3390/cells15131224

**Published:** 2026-07-06

**Authors:** Perla Ugalde-Muñiz, Yetzalen Olvera-Valderrabano, Rafael Lugo-Huitrón, Abraham Landa, Luz Navarro

**Affiliations:** 1 Departamento de Fisiología, Facultad de Medicina, Universidad Nacional Autónoma de México, Mexico City 04510, Mexico; perlifa@hotmail.com (P.U.-M.); yet.olvera@ciencias.unam.mx (Y.O.-V.); rafaluahu@gmail.com (R.L.-H.); 2 Departamento de Microbiología y Parasitología, Facultad de Medicina, Universidad Nacional Autónoma de México, Mexico City 04510, Mexico; landap@unam.mx

**Keywords:** D2 dopamine receptor, quinpirole, REM sleep deprivation, neuroinflammation, microglia, NF-κB translocation, hippocampus

## Abstract

**Highlights:**

**What are the main findings?**

- REM sleep deprivation (RSD) induces an increase in Iba-1 expression and NF-κB nuclear expression in the hippocampus and cortex.
- Systemic administration of quinpirole (QUIN), a D2-like dopamine receptor, reduces Iba-1 expression, reverses morphological changes, and reduces NF-κB nuclear expression in microglia.

**What are the implications of the main findings?**

- D2 receptor activation exerts an anti-inflammatory effect on microglia during REM sleep loss.
- The dopaminergic system represents a potential therapeutic target for neuroinflammation associated with sleep disturbances.

**Abstract:**

Sleep deprivation is a recognized risk factor for neuroinflammatory and neurodegenerative disorders. Dopamine signaling through D2 receptors (DRD2) has emerged as a potential immunomodulatory pathway in the central nervous system. The present study investigated whether activation of DRD2 by quinpirole (QUIN) modulates astrocytic and microglial responses and NF-κB nuclear translocation in a murine model of rapid eye movement sleep deprivation (RSD). Male CD1 mice were subjected to 72 h of RSD and treated with QUIN (2 mg/kg/day). GFAP, Iba-1, and NF-κB expression were evaluated in hippocampal subregions (CA1, CA3, dentate gyrus) and the medial parietal cortex using immunofluorescence and confocal microscopy. RSD increased GFAP and Iba-1 expression and induced morphological changes consistent with glial activation. Notably, RSD increased NF-κB nuclear expression in microglia. QUIN administration reduced Iba-1 expression, attenuated microglial morphological alterations, and reduced NF-κB nuclear expression across all analyzed regions, even in RSD-subjected mice. These findings suggest that DRD2 activation exerts anti-inflammatory effects in the brain during REM sleep deprivation and that dopaminergic signaling may represent a key target for neuroinflammation associated with sleep loss.

## 1. Introduction

Sleep plays a key role in neuron repair. Nowadays, society commonly experiences restricted sleep periods, interrupted sleep, or low-quality sleep. Each of these conditions is recognized as a risk factor for developing neurodegenerative disorders [1]. Sleep must comprise two stages: rapid eye movement (REM) sleep and non-rapid eye movement (NREM) sleep [2]. Although it is an extremely complex physiological process involving neuronal activity and neurotransmitter modulation, dopamine (DA) and dopaminergic circuits are crucial for REM induction and control [3,4].

It has been reported that both acute and chronic sleep loss alter immune responses, mainly through aberrant or increased cell activation and the exacerbated release of proinflammatory molecules, such as tumor necrosis factor alpha (TNF-α), interleukin-1β (IL-1β), IL-17, IL-6, and C-reactive protein [5]. In this context, increased levels of TNFα, IL-1β, and IL-6 impact the integrity of tight junctions present in the blood–brain barrier (BBB) by decreasing expression of ocludin-1 and zona ocludens-1 (ZO-1); such alterations promote cytokines and peripheral immune cells to both invade the central nervous system tissue [6].

Additionally, previous work has shown that total sleep deprivation increases IL-6 and overexpression of the ionized calcium-binding adapter molecule 1 (Iba-1) in the hippocampus, the latter being a marker of microglial activation [7].

Recently, it has been reported that DA can induce the proliferation, differentiation, and activation of immune cells [8]. Although several molecular mechanisms have been identified in neuroimmunomodulation, the immunomodulatory role of DA has recently attracted attention. When activated macrophages are exposed to DA, IFN-γ release increases, indirectly enhancing their phagocytic activity [9]. On the other hand, Haskó and coworkers showed in this cell type that D2 receptor (DRD2) agonists, such as bromocriptine and quinpirole (QUIN), reduce TNF-α and nitric oxide (NO) release after macrophages were treated with lipopolysaccharide (LPS) [10].

The anti-inflammatory effects of DRD2 were explored in the peripheral immune system [11] as well as in the central nervous system (CNS) [12,13], where microglia are considered the axis of such regulation since these cells express relatively high levels of D1 and D2 DA receptors [14], strongly suggesting the association of both systems in neural tissue. Further, Yoshioka and coworkers (2020) [15] showed in microglial cell cultures that, upon LPS exposure, DA modulates the release of proinflammatory cytokines by inhibiting NF-κB p65 translocation to the nucleus.

Dopaminergic agonists are an effective tool for understanding the effects of DA on inflammation. QUIN is commonly used in such models since it rapidly crosses the BBB and is detectable in cerebrospinal fluid (CSF) when intravenously administered from 0.17 to 0.86 mg/kg in rats [16]. Regarding QUIN effects, Alam and coworkers demonstrated that intraperitoneal administration of 1 mg/kg QUIN prevented neuronal damage, glial activation, and neuroinflammation after traumatic brain injury (TBI). Interestingly, such protection occurs through the DRD2 and the Akt/GSK-β pathway [17].

Previously, our workgroup demonstrated that systemic administration of QUIN reduced both systemic and hippocampal TNFα, IL-6, and IL-1β levels induced by 72 h of REM sleep deprivation (RSD) in male adult CD1 mice [18]. These findings demonstrate the fundamental role of DRD2 in RSD-induced proinflammatory responses. Thus, the present study aimed to explore, within the same RSD model, whether activation of DRD2 by QUIN influences astrocyte and microglial responses and nuclear NF-κB expression.

## 2. Materials and Methods

### 2.1. Experimental Animals

All experiments were performed with male CD1 (ICR) 8-week-old mice (only male mice were used to reduce hormonal variability). Animals were maintained under standard conditions on a 12:12 light–dark cycle (lights on at 7:00 a.m. and off at 7:00 p.m.). Water and food were provided ad libitum at room temperature (22 °C).

### 2.2. Ethical Considerations

Every surgical and pain-inducing procedure was performed using anesthetics in animals in accordance with the Norma Oficial Mexicana NOM-062-ZOO-1999 [19]. The animals were obtained from the Bioterium Unit of the Faculty of Medicine at the National Autonomous University of Mexico. All experimental procedures were approved by the Ethics Committee of the Faculty of Medicine of the National Autonomous University of Mexico (UNAM) (project: FM/DI/078/2022; approved on 6 September 2022).

### 2.3. Treatments

Animals were randomly assigned to four experimental groups of five subjects each. The control group was administered 80 µL of saline solution (i.p.) for 3 consecutive days. The agonist QUIN was used to specifically activate DRD2. The QUIN group received quinpirole hydrochloride (Sigma Aldrich, St. Louis, MO, USA) for 3 days (2 mg/kg/day). Such a dose was selected based on our previous results, in which proinflammatory cytokines were downregulated in the hippocampus [18].

**Figure 1:**
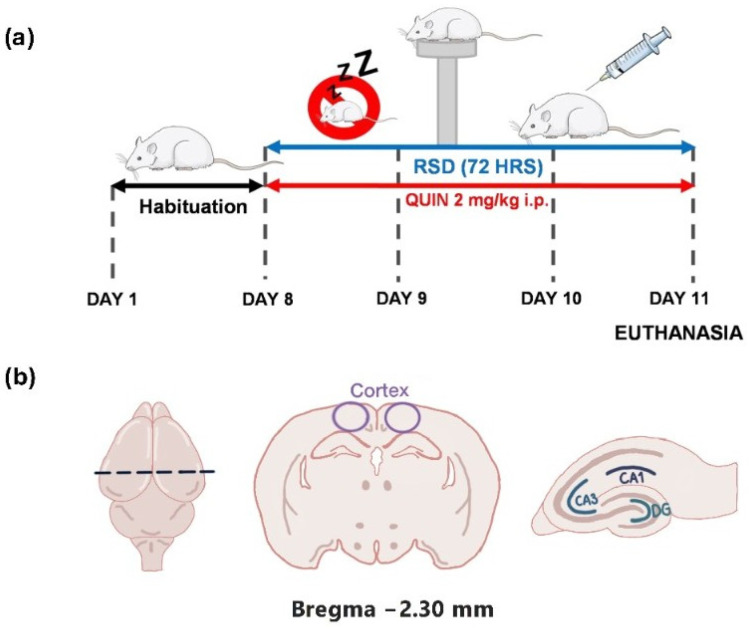
(**a**) Timeline showing the RSD model and QUIN administration. After one week of habituation in the vivarium, mice were subjected to the corresponding pharmacological treatments. On day 11, animals were euthanized for tissue dissection. (**b**) The histological slices are represented, highlighting the analyzed regions: medial parietal cortex (CTX), CA1, CA3, and dentate gyrus (DG) from the hippocampus (ML = 1.2 mm, AP = −2.18 mm, and DV = −1.52 mm from Bregma).

RSD was induced by the multiple platforms box for 72 h. Animals moved freely on the platforms, and water was replaced daily. The last group was subjected to RSD and treated with QUIN in parallel (RSD+QUIN) (Figure 1). At the end of the treatments, animals were euthanized with 50 mg/kg sodium pentobarbital. For immunofluorescence tests, mice were perfused with saline solution, then with 4% paraformaldehyde. Brains were recovered and cryopreserved, embedded in 30% sucrose at 4 °C.

### 2.4. Immunofluorescence for GFAP, Iba-1, and NF-κB

Brains were sectioned using a Leica CM1100 cryostat (Nussloch, Germany)to obtain 20 μm and 40 μm coronal slices for epifluorescence and confocal analysis, respectively. Images were captured from CTX, CA1, CA3, and DG. The coordinates for the identification of the medial parietal cortex CTX, CA1, CA3, and dentate gyrus DG (ML = 1.2 mm, AP = −2.18 mm, and DV = −1.52 mm from Bregma) were established by using the Paxinos Mouse Brain Atlas. All slices were kept in phosphate-buffered saline (PBS) containing 137 mM NaCl, 10 mM HPO_4_, and 2.7 mM KCl, with pH 7.4. For each experimental group, three consecutive slices per mouse were used for testing and analysis. All slices were incubated in Diva Decloaker solution (Biocare Medical, Pacheco, CA, USA) for antigen retrieval in a hot water bath for 7 min. After cooling to room temperature, samples were washed with PBS. To permeabilize cell membranes and block nonspecific antigens, the samples were incubated with 0.2% Triton X-100 and blocking buffer (bovine serum albumin 4%) for 1 h at room temperature. The slices underwent three washing cycles before primary antibody incubation.

To analyze GFAP and Iba-1 expression, epifluorescence immunostainings were performed. Brain slices were incubated with polyclonal anti-GFAP host Rabbit (Genetex GTX108711; Hsinchu, Taiwan) at 1:200 for 1 h at room temperature and overnight at 4 °C. After three washes, the samples were incubated with an Alexa 594-coupled polyclonal secondary antibody host Goat (Genetex GTX213110-05; 1:700; Hsinchu, Taiwan) for 3 h. An additional set of slices was used for Iba-1 immunodetection with 1:300 polyclonal anti-Iba-1 host Rabbit (Genetex GTX100042; Hsinchu, Taiwan), both incubated for 1 h at room temperature, then overnight at 4 °C. After three washes, the samples were incubated with an Alexa 594-coupled polyclonal secondary antibody host Goat (Genetex GTX213110-05; 1:700; Hsinchu, Taiwan) for 3 h. After eight washing cycles, 250 μL of DAPI was added for nuclei staining for 5 min, followed by two additional washes. Then, the slices were mounted on slides using Vectashield (H-1000, Vector) (Newwark, CA, USA) as the mounting medium.

The Iba-1 and GFAP immunofluorescence images were captured on a Nikon ECLIPSE Ci epifluorescence microscope (Yokohama, Japan) at 40× under the following conditions: the room was used exclusively for microscopy, with controlled dim red light for each observation, thereby preserving the integrity of the fluorochromes and avoiding photobleaching of the samples. When the fields and areas of interest were delimited, the slices were examined in the corresponding fluorescence channels. For image digitization, a Canon T7i DSLR camera (Oita, Japan) was directly attached to the ocular via an adapter that substitutes for the camera lens; thus, the sensor directly captures the images observed by the ocular, maintaining the camera’s stability. The digitalization parameters were homologated for each image at an exposure time of 15 s and ISO 500.

Additionally, for detailed NF-κB cellular localization, samples were analyzed with a Leica TCS-SP8 confocal microscope (Manneheim, Germahy) (63×). A set of slices was used for double immunodetection with 1:300 Recombinant Monoclonal anti-Iba-1 host Rat (Thermo Fisher HL22-RT, Rockford, IL, USA) and 1:200 polyclonal anti-NF-κB p65 host Rabbit (Genetex GTX133899, Hsinchu, Taiwan), both incubated for 1 h at room temperature, then overnight at 4 °C. After three rounds of washes, samples were incubated with the secondary antibodies Anti-rabbit IgG Alexa Fluor 488 Conjugate (Cell Signaling 4412, Beverly, MA, USA), host goat and Anti-rat IgG Alexa Fluor 555 Conjugate (Cell Signaling 4417, Beverly, MA, USA) host goat, both diluted 1:500. After eight washing cycles, 250 μL of DAPI were added for nuclei staining for 5 min, followed by two additional washes. Then, the slices were mounted on slides using Vectashield (H-1000, Vector, Newwark, CA, USA) as the mounting medium.

### 2.5. Mean Fluorescence Intensity Analysis for Iba-1, GFAP, and NF-κB Immunostainings

To evaluate Iba-1 and GFAP expression in slices, all digital images were analyzed with the open-access FIJI software version 2.16.0. Two images per mouse were included in this analysis; thus, a total of 10 images were processed for each experimental group. Images were imported into the software and converted to grayscale using the Image > Type > 8 bits option. Then, the threshold was set in the menu Image > AdjustZ > Threshold, and applied when the cells’ morphology was highly defined.

For fluorescence quantification, the Analyze > Measure function was used, with the Mean parameter, while the Area function represents the relative intensity per cell. For each brain region, individual cells were selected and circled to establish an area around them. The measure was executed with the menu Analyze > Measure. This method was repeated for each cell. A total of 25 cells were analyzed in the CA1, CA3, DG, and CTX regions across the experimental groups. The determination of GFAP- and Iba-1-positive cells was guided by four criteria: (1) cells were randomly selected in each field; (2) cells must not overlap each other; (3) the entire cell body and ramifications must be visible inside the field; and (4) both nuclei and ramifications are clearly distinguished. For testing and analysis, three slices per mouse were obtained for each experimental group.

The nuclear localization of NF-κB was determined as previously described; however, the circled areas comprised only DAPI-positive nuclei. For NF-κB quantification, a total of 20 cells were analyzed in the CA1, CA3, DG, and CTX regions across the experimental groups.

### 2.6. Colocalization Analysis of Iba-1 and NF-κB in Confocal Microscopy Images

Nuclear translocation of NF-κB in microglial cells was assessed by colocalization of Iba-1 and DAPI markers. Confocal microscopy images were loaded into their respective channels in FIJI. Subsequently, the Color > Merge Channels function was selected in the dialog box to assign each image to its corresponding channel. Then, a montage was created using the Image > Stack > Make a Montage option. All parameters were configured according to the image characteristics. Finally, the three channels (Iba-1, NF-κB, and DAPI) were merged using Image > Stacks > Tools > Magic Montage Tools > Select Panels Tool, which enabled us to assess marker colocalization.

### 2.7. Statistical Analysis

All statistical analyses were performed using GraphPad Prism 9 (GraphPad Software, San Diego, CA, USA). Data are presented as mean ± standard error of the mean (SEM). Homogeneity of variances was assessed using Brown–Forsythe and Bartlett’s tests. When the assumptions of normality and homogeneity of variances were met, a nested one-way ANOVA was conducted, followed by Tukey’s post hoc test for multiple comparisons. A nested one-way ANOVA design was used because five cells per experimental subject were analyzed (technical replicates), with five subjects per group (biological replicates).

## 3. Results

### 3.1. Effect of RSD and QUIN Administration on GFAP Expression in Astrocytes

**Figure 2:**
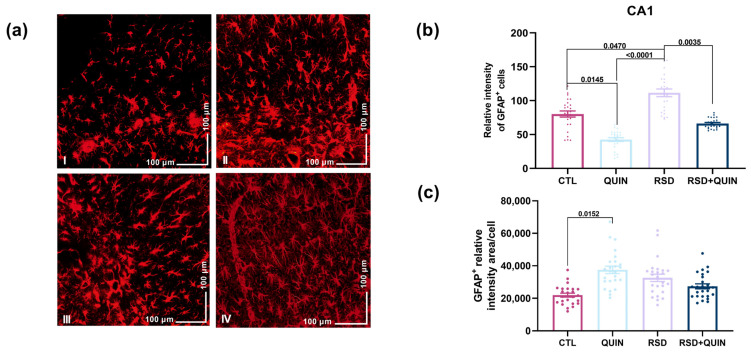
Effect of QUIN administration (2 mg/kg/day for three days; i.p.) along with the RSD in GFAP expression in CA1 from hippocampus in mice. (**a**) Representative images (40×) of GFAP immunofluorescence from experimental groups: (**I**) CTL, (**II**) QUIN, (**III**) RSD, and (**IV**) RSD+QUIN. (**b**) Mean fluorescence intensity of GFAP-marked cells. (**c**) Relative fluorescent area of GFAP+ per cell. All values were obtained with FIJI software; data are shown as mean ± SEM (*n* = 5). Individual dots represent one cell; a total of five cells per animal were analyzed, and five subjects were included for each experimental group. Statistical analysis was performed by a nested one-way ANOVA test, followed by Tukey’s test (*p* < 0.05).

**Figure 3:**
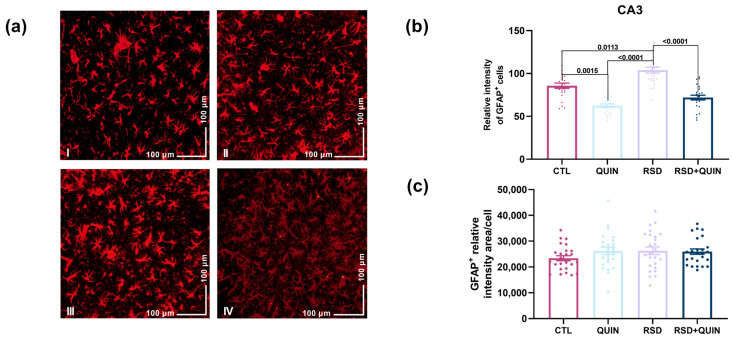
Effect of QUIN administration alone and along with RSD in CA3 from the hippocampus. (**a**) Representative images (40×) of GFAP immunofluorescence from experimental groups: (**I**) CTL, (**II**) QUIN, (**III**) RSD, and (**IV**) RSD+QUIN. (**b**) Mean fluorescence intensity of GFAP-marked cells. (**c**) Relative fluorescent area of GFAP+ per cell. All values were obtained with FIJI software; data are shown as mean ± SEM (*n* = 5). Individual dots represent one cell; a total of five cells per animal were analyzed, and five subjects were included for each experimental group. Statistical analysis was performed by a nested one-way ANOVA test, followed by Tukey’s test (*p* < 0.05).

**Figure 4:**
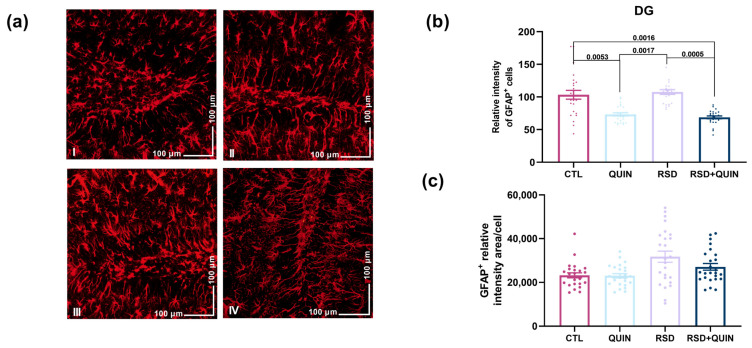
Effect of QUIN administration alone and in combination with RSD on DG from the hippocampus. (**a**) Representative images (40×) of GFAP immunofluorescence from experimental groups: (**I**) CTL, (**II**) QUIN, (**III**) RSD, and (**IV**) RSD+QUIN. (**b**) Mean fluorescence intensity of GFAP-marked cells. (**c**) Relative fluorescent area of GFAP+ per cell. All values were obtained with FIJI software; data are shown as mean ± SEM (*n* = 5). Individual dots represent one cell; a total of five cells per animal were analyzed, and five subjects were included for each experimental group. Statistical analysis was performed using a nested one-way ANOVA, followed by Tukey’s test (*p* < 0.05).

**Figure 5:**
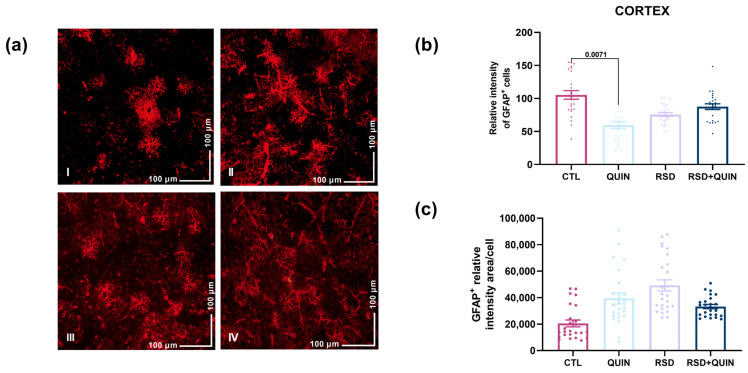
Effect of QUIN administration alone and along with RSD in CTX. (**a**) Representative images (40×) of GFAP immunofluorescence from experimental groups: (**I**) CTL, (**II**) QUIN, (**III**) RSD, and (**IV**) RSD+QUIN. (**b**) Mean fluorescence intensity of GFAP-marked cells. (**c**) Relative fluorescent area of GFAP+ per cell. All values were obtained with FIJI software; data are shown as mean ± SEM (*n* = 5). Individual dots represent one cell; a total of five cells per animal were analyzed, and five subjects were included for each experimental group. Statistical analysis was performed using a nested one-way ANOVA, followed by Tukey’s test (*p* < 0.05).

This work aimed to determine whether DRD2 activation modulates neuroinflammation associated with RSD. Therefore, we first evaluated astrocyte activation by immunofluorescence using GFAP. We found that QUIN treatment significantly decreased GFAP expression compared with the CTL group in CA1 (*p* = 0.0145), CA3 (*p* = 0.0015), DG (*p* = 0.0053), and CTX (*p* = 0.0071) (Figure 2b, Figure 3b, Figure 4b and Figure 5b). On the other hand, RSD significantly increased GFAP expression compared to the CTL group in CA1 (*p* = 0.0470) and CA3 (*p* = 0.0113) (Figure 2b and Figure 3b). No significant differences were found in DG and CTX (Figure 4b and Figure 5b). Regarding the QUIN effect, when this agonist was administered to RSD mice, a significantly decreased GFAP expression was found vs. the RSD animals across all three hippocampus regions: CA1 (*p* = 0.0035), CA3 (*p* < 0.0001), and DG (*p* = 0.0005) (Figure 2b, Figure 3b and Figure 4b). However, in CTX, no differences were observed between the RSD group and RSD+QUIN (Figure 5b). Furthermore, when the GFAP-positive cell area was measured, the RSD group did not show significant changes compared with the CTL group. Interestingly, the astrocytes responded to QUIN by increasing the cell area only in CA1 when compared to CTL (*p* = 0.0152) (Figure 2c). Taken together, these findings indicate that RSD induces changes in GFAP expression; however, the response appears to differ between the cortex and the hippocampus. Interestingly, QUIN administration alone can reduce GFAP expression in all regions. Nonetheless, despite GFAP expression data, no effect was observed in the cellular area of astrocytes from RSD and RSD+QUIN animals.

### 3.2. Effect of RSD and QUIN Administration on Microglial Activation

Since our results indicate differential astrocitic response to RSD and QUIN in the cortex and hippocampus, we aimed to examine whether DRD2 activation also regulates microglia, a critical cell population in the CNS that participates in immune response.

To this end, immunofluorescence against Iba-1 was performed to evaluate microglial activation during RSD and QUIN administration.

**Figure 6:**
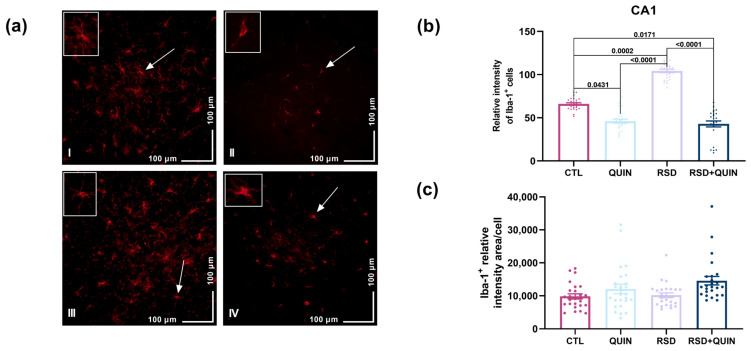
Effect of QUIN administration, RSD, and RSD+QUIN on Iba-1 expression in CA1. (**a**) Representative immunofluorescence images of Iba-1 fluorescence mark at 40× magnification under the experimental conditions: (**I**) CTL, (**II**) QUIN, (**III**) RSD, and (**IV**) RSD+QUIN; insets show magnification of microglial cells (white arrows) for detailed visualization of morphological changes according to treatment. (**b**) Mean fluorescence intensity values of Iba-1 in CA1 across the four experimental groups. (**c**) Relative fluorescent area of Iba-1+ per cell. All values were obtained with FIJI software and are shown as mean ± SEM (*n* = 5). Individual dots represent one cell; a total of five cells per animal were analyzed, and five subjects were included for each experimental group. Statistical analysis was performed using a nested one-way ANOVA, followed by Tukey’s test (*p* < 0.05).

**Figure 7:**
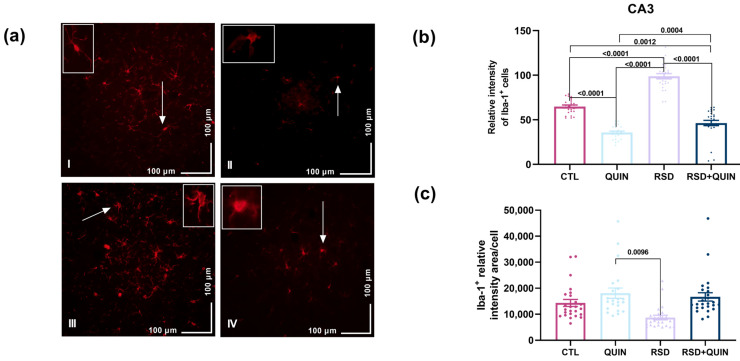
Effect of QUIN administration, RSD, and RSD+QUIN on Iba-1 expression in CA3. (**a**) Representative immunofluorescence images of Iba-1 fluorescence mark at 40× magnification under the experimental conditions: (**I**) CTL, (**II**) QUIN, (**III**) RSD, and (**IV**) RSD+QUIN; insets show magnification of microglial cells (white arrows) for detailed visualization of morphological changes according to treatment. (**b**) Mean fluorescence intensity values of Iba-1 in CA3 across the four experimental groups. (**c**) Relative fluorescent area of Iba-1+ per cell. All values were obtained using FIJI software and are shown as mean ± SEM (*n* = 5). Individual dots represent one cell; a total of five cells per animal were analyzed, and five subjects were included for each experimental group. Statistical analysis was performed using a nested one-way ANOVA, followed by Tukey’s test (*p* < 0.05).

**Figure 8:**
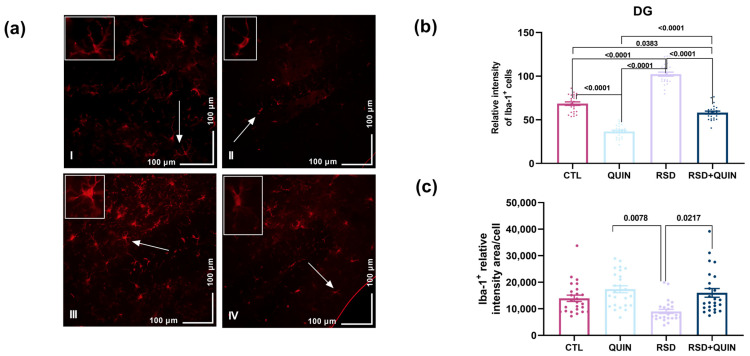
Effect of QUIN administration, RSD, and RSD+QUIN on Iba-1 expression in DG. (**a**) Representative immunofluorescence images of Iba-1 fluorescence mark at 40× magnification under the experimental conditions: (**I**) CTL, (**II**) QUIN, (**III**) RSD, and (**IV**) RSD+QUIN; insets show magnification of microglial cells (white arrows) for detailed visualization of morphological changes according to treatment. (**b**) Mean fluorescence intensity values of Iba-1 in DG across the four experimental groups. (**c**) Relative fluorescent area of Iba-1+ per cell. All values were obtained using FIJI software and are shown as mean ± SEM (*n* = 5). Individual dots represent one cell; a total of five cells per animal were analyzed, and five subjects were included for each experimental group. Statistical analysis was performed using a nested one-way ANOVA, followed by Tukey’s test (*p* < 0.05).

**Figure 9:**
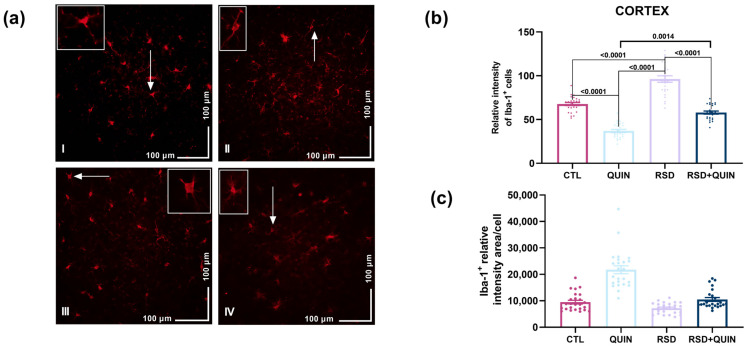
Effect of QUIN administration, RSD, and RSD+QUIN on Iba-1 expression in the CTX. (**a**) Representative immunofluorescence images of Iba-1 fluorescence mark at 40× magnification under the experimental conditions: (**I**) CTL, (**II**) QUIN, (**III**) RSD, and (**IV**) RSD+QUIN; insets show magnification of microglial cells (white arrows) for detailed visualization of morphological changes according to treatment. (**b**) Mean fluorescence intensity values of Iba-1 in the CTX across the four experimental groups. (**c**) Relative fluorescent area of Iba-1+ per cell. All values were obtained using FIJI software and are shown as mean ± SEM (*n* = 5). Individual dots represent one cell; a total of five cells per animal were analyzed, and five subjects were included for each experimental group. Statistical analysis was performed using a nested one-way ANOVA, followed by Tukey’s test (*p* < 0.05).

The results show that QUIN significantly decreased the relative intensity of Iba-1 compared with the CTL group in CA1 (*p* = 0.0431), CA3 (*p* < 0.0001), GD (*p* < 0.0001), and CTX (*p* < 0.0001) (Figure 6b, Figure 7b, Figure 8b and Figure 9b). In contrast, RSD significantly increased Iba-1 expression compared to the CTL group in CA1 (*p* = 0.002), CA3 (*p* < 0.0001), DG (*p* < 0.0001), and CTX (*p* < 0.0001) (Figure 6b, Figure 7b, Figure 8b and Figure 9b). On the other hand, in the RSD+QUIN group, QUIN administration significantly decreased Iba-1 expression relative to the RSD group in CA1 (*p* < 0.0001), CA3 (*p* < 0.0001), DG (*p* < 0.0001), and CTX (*p* < 0.0001). We also found a decrease in Iba-1 expression in the RSD group compared to the CTL group in CA1 (*p* = 0.0171) and CA3 (*p* = 0.0012) (Figure 6b and Figure 7b).

Moreover, when the area of microglia was evaluated (Iba-1+ cells), a significant reduction was observed in the CA3 (*p* = 0.0096) and DG (*p* = 0.0078) from RSD animals compared to the QUIN group (Figure 7c and Figure 8c). Furthermore, a descriptive analysis was conducted to evaluate microglial morphology (soma area, length, number, and branch complexity).

Control cells from CA1, CA3, DG, and CTX exhibited characteristic morphology of the inactive state, featuring elongated, small soma and multiple long, thin processes (Figure 6(aI), Figure 7(aI), Figure 8(aI) and Figure 9(aI)). On the other hand, the QUIN group showed morphologies similar to those of the CTL, with small, elongated soma in all regions. However, these cells showed a lower ramification density, yet remained long and thin (Figure 6(aII), Figure 7(aII), Figure 8(aII) and Figure 9(aII)).

In the RSD group, an ameboid morphology was observed in microglia from all regions (Figure 6(aIII), Figure 7(aIII), Figure 8(aIII) and Figure 9(aIII)), with larger somas and fewer ramifications that appeared shorter and thicker than those in the CTL group. Finally, the animals subjected to RSD and QUIN administration showed evident changes in microglial morphology in every region (Figure 6(aIV), Figure 7(aIV), Figure 8(aIV) and Figure 9(aIV)), with smaller somas. The ramification density was equal to that of the RSD group; however, contrary to this group, these ramifications seemed longer and thinner.

### 3.3. Nuclear Expression of NF-κB in Microglia After RSD and QUIN Administration

**Figure 10:**
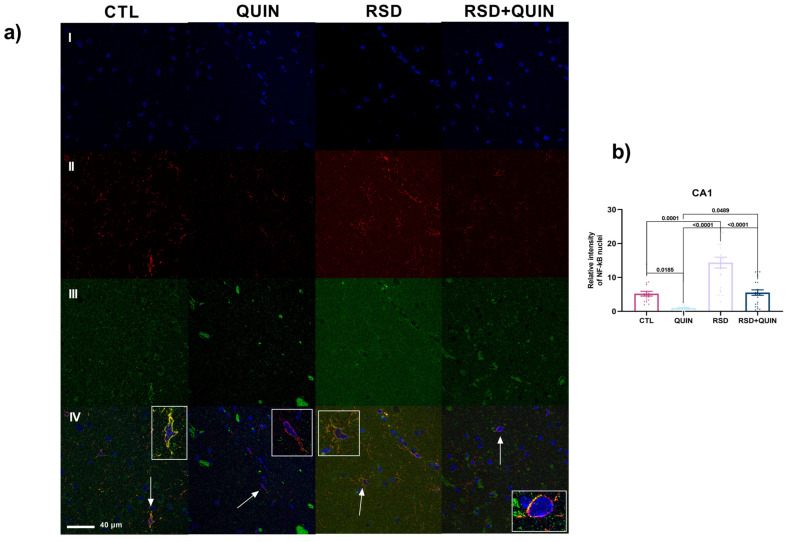
Effect of QUIN administration on nuclear translocation of NF-κB from CA1 in all experimental groups. (**a**) Representative confocal microscopy images of NF-κB immunofluorescence at 63×; boxes show magnification of indicated cells (white arrows) for detailed visualization of nuclear signal. Marks are shown as: (**I**) DAPI, (**II**) Iba-1, (**III**) NF-κB, and (**IV**) Merge. (**b**) Mean fluorescence intensity measures of NF-κB mark for every experimental group. Values were obtained using FIJI software; data are shown as mean ± SEM (*n* = 5). Individual dots represent one cell; a total of four cells per animal were analyzed, and five subjects were included for each experimental group. Statistical analysis was performed using a nested one-way ANOVA, followed by Tukey’s test (*p* < 0.05).

The data show that astrocyte response differs between the cortex and hippocampus and is variable across experimental groups, whereas Iba-1 exhibits a similar pattern across treatments and regions. Based on these observations, we aimed to test, using immunostainings, whether nuclear NF-κB expression increases in microglia in all regions. First, the NF-κB mark was significantly lower in QUIN group mice compared to CTL mice in CA1 (*p* = 0.0185) (Figure 10b).

**Figure 11:**
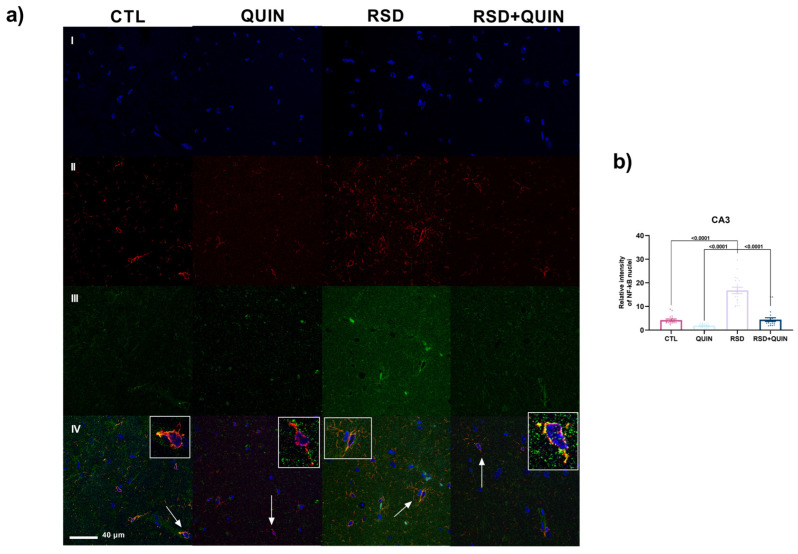
Effect of QUIN administration on nuclear translocation of NF-κB from CA3 in all experimental groups. (**a**) Representative confocal microscopy images of NF-κB immunofluorescence at 63×; boxes show magnification of indicated cells (white arrows) for detailed visualization of nuclear signal. Marks are shown as: (**I**) DAPI, (**II**) Iba-1, (**III**) NF-κB, and (**IV**) Merge. (**b**) Mean fluorescence intensity measures of NF-κB mark for every experimental group. Values were obtained using FIJI software; data are shown as mean ± SEM (*n* = 5). Individual dots represent one cell; a total of four cells per animal were analyzed, and five subjects were included for each experimental group. Statistical analysis was performed using a nested one-way ANOVA, followed by Tukey’s test (*p* < 0.05).

**Figure 12:**
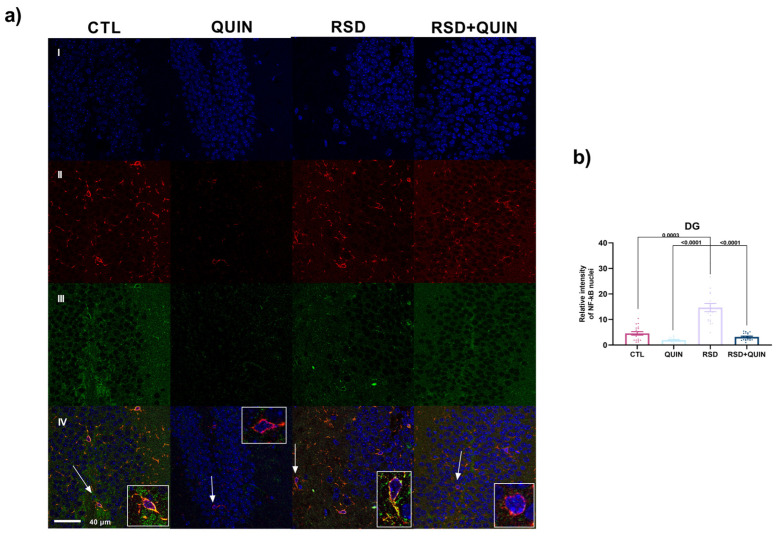
Effect of QUIN administration on nuclear translocation of NF-κB from DG in all experimental groups. (**a**) Representative confocal microscopy images of NF-κB immunofluorescence at 63×; boxes show magnification of indicated cells (white arrows) for detailed visualization of nuclear signal. Marks are shown as: (**I**) DAPI, (**II**) Iba-1, (**III**) NF-κB, and (**IV**) Merge. (**b**) Mean fluorescence intensity measures of NF-κB mark for every experimental group. Values were obtained using FIJI software; data are shown as mean ± SEM (*n* = 20). Individual dots represent one cell; a total of four cells per animal were analyzed, and five subjects were included for each experimental group. Statistical analysis was performed using a nested one-way ANOVA test, followed by Tukey’s test (*p* < 0.05).

**Figure 13:**
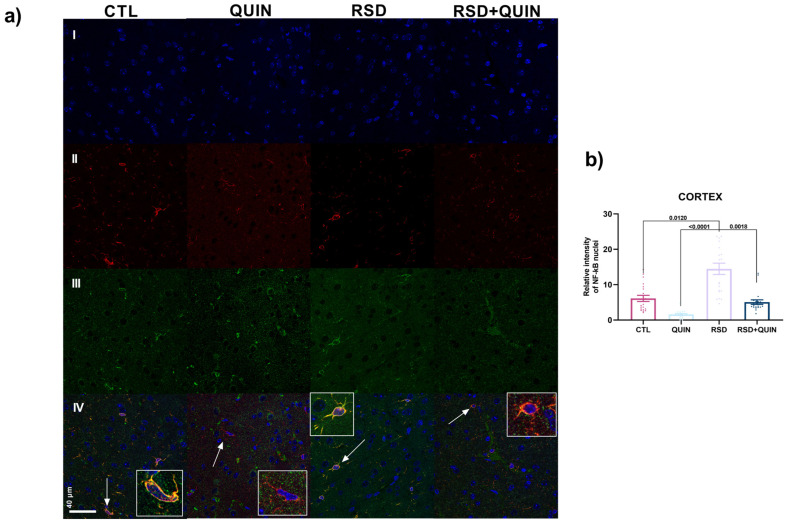
Effect of QUIN administration on nuclear translocation of NF-κB from the CTX in all experimental groups. (**a**) Representative confocal microscopy images of NF-κB immunofluorescence at 63×; boxes show magnification of indicated cells (white arrows) for detailed visualization of nuclear signal. Marks are shown as: (**I**) DAPI, (**II**) Iba-1, (**III**) NF-κB, and (**IV**) Merge. (**b**) Mean fluorescence intensity measures of NF-κB mark for every experimental group. Values were obtained using FIJI software; data are shown as mean ± SEM (*n* = 20). Individual dots represent one cell; a total of four cells per animal were analyzed, and five subjects were included for each experimental group. Statistical analysis was performed using a nested one-way ANOVA, followed by Tukey’s test (*p* < 0.05).

Conversely, the NF-κB expression was increased in the RSD group vs. the CTL group in CA1 (*p* < 0.0001), CA3 (*p* < 0.0001), DG (*p* = 0.0003), and CTX (*p* = 0.0120) (Figure 10b, Figure 11b, Figure 12b and Figure 13b), and compared to QUIN in CA1 (*p* < 0.0001) (Figure 10b), CA3 (*p* < 0.0001) (Figure 11b), DG (*p* < 0.0001) (Figure 12b), and CTX (*p* < 0.0001) (Figure 13b). The RSD, along with QUIN injections, reduced the NF-κB expression with respect to RSD in all regions: CA1 (*p* < 0.0001), CA3 (*p* <0.0001), DG (*p* < 0.0001), and CTX (*p* = 0.0018) (Figure 10b, Figure 11b, Figure 12b and Figure 13b). Finally, this same group (RSD+QUIN) also reduced the NF-κB nuclear expression vs. the QUIN group in CA1 (*p* < 0.0489) (Figure 10b). Our data suggest that RSD induces NF-κB nuclear translocation in microglia; additionally, QUIN decreases this translocation, even when mice were subjected to RSD. Such an effect is conserved in all analyzed regions.

## 4. Discussion

### 4.1. Effect of Sleep Deprivation and QUIN Administration on Astrocyte Activation

In this study, we show that sleep deprivation increases GFAP and Iba-1 expression, consistent with increased astrocyte and microglial reactivity in the mouse hippocampus. Furthermore, systemic quinpirole treatment during sleep deprivation prevented these cellular changes.

Sleep deprivation induces robust inflammation across the CNS by stimulating the production of proinflammatory cytokines, such as IL-1β, IL-6, and TNF-α, in various brain regions, including the hippocampus [18,20,21,22]. Astrocytes and microglia become reactive when stimulated by pro-inflammatory cytokines, thus amplifying the response. In this context, GFAP is widely recognized as a classical marker of astrocytic activation, as its expression is directly related to inflammatory or noxious stimuli [23].

Our results indicate that the astrocytic response to RSD in CA1 and CA3 differs from that in DG (Figure 2b, Figure 3b and Figure 4b). Nonetheless, this contrasts with previous findings from a different study, which showed that 20 h/day of REM sleep restriction for 14 days decreased GFAP expression in the hippocampus [24]. Notably, the temporal pattern of sleep restriction influences astrocyte responses. These cells possess highly plastic capacities when brain tissue is exposed to an inflammatory stimulus. Even if GFAP expression increases and cells develop a reactive phenotype in the early stages of inflammation, astrocytes may return to a homeostatic state as cytokines are gradually removed, depending on the intensity of the inflammatory stimulus [25].

Therefore, the RSD period is a determining factor in activating these cells, and it evidently has complex, dynamic regulation over time. A transition may occur through different degrees of activation rather than remaining static across regions. Which may underlie the differential response among hippocampal regions. Interestingly, numerous studies using different RSD models report increased GFAP expression, indicating astrocyte activation in the hippocampus, specifically the CA1 and DG [22,26,27,28]. Such evidence reinforces our results; therefore, we consider astrocytic activation as a key regulator of the neuroinflammatory mechanisms associated with RSD. Previous studies have reported that DRD2 knockout mice exhibit robust inflammatory responses and increased GFAP expression in multiple brain regions, indicating that activation of these receptors is key for immunomodulating reactive astrogliosis [29].

We also sought to explore the effect of QUIN, a D2DR agonist, in mice with regular sleep periods who were then subjected to RSD. As expected, QUIN downregulated GFAP expression in the hippocampus, indicating that astrocytes become hypo-responsive or maintain their homeostatic state in this region (Figure 2, Figure 3 and Figure 4). It was previously reported that QUIN suppresses GFAP expression.

This effect has been reported in vitro, where cell cultures incubated with QUIN and MPTP (1-methyl-4-phenyl-1,2,3,6-tetrahydropyridine) decreased the GFAP+ astrocytes via αB-crystallin (CRYAB) [29]. An analogous molecular mechanism probably underlies the effect on the hippocampus, which would explain the reduced GFAP expression observed in our RSD+QUIN model. Hence, DRD2 activation has been proposed as a therapeutic target, as it reduces astrocytic reactivity in the brain cortex, even in the face of highly inflammatory insults such as TBI [17]. In contrast, our results in the CTX showed that QUIN did not exert such a modulatory effect. However, it is worth noting that only a fraction of the CTX was analyzed. The cortical tissue is highly heterogeneous and complex, with a cytoarchitecture and physiology that warrant further exploration.

### 4.2. Effect of Sleep Deprivation and QUIN Administration on Microglial Activation

Given the increased astrogliosis, we sought to investigate the effect of RSD on microglia. The response of microglial cells is essential for regulating neuroinflammation; upon an inflammatory stimulus, they become reactive, altering their morphology and releasing a set of molecules, mostly proinflammatory cytokines, depending on the activation phenotype. Different methods to evaluate microglial activation have been proposed; one of the most widely used is based on Iba-1 expression. Iba-1 overexpression is involved in structural changes in the cytoskeleton and cell membrane, enabling cell movement, migration, and phagocytosis [30]. We sought to evaluate Iba-1 in our model; the data indicate that RSD increased Iba-1 fluorescence intensity compared to the CTL group in CA1, CA3, DG, and the CTX (Figure 6, Figure 7, Figure 8 and Figure 9). RSD models induce microglia activation and increase Iba-1 expression in the hippocampus and cortex [22,27,28]. Systemic QUIN administration reduced Iba-1 fluorescence intensity, even below the control levels in CA1, CA3, DG, and the CTX. This was not surprising given the GFAP expression data. Analysis of the hippocampus and cortex of mice subjected to RSD+QUIN revealed reduced Iba-1 expression (Figure 6a,b, Figure 7a,b, Figure 8a,b and Figure 9a,b). It is worth noting that, under our RSD model, QUIN has the same effect across all regions evaluated, decreasing Iba-1 expression, even in DG, where QUIN did not affect GFAP expression in astrocytes. This raises a new question: Does the DG require specific conditions to trigger astrogliosis or a microglial response? This question remains unsolved with our results.

The RSD-derived morphological manifestations in microglia include a reduction in the number of ramifications, shortened endings and processes, and shorter microglial cell length [31]. Surprisingly, we did not observe significant differences in microglial area; however, these cells appeared to adopt an active morphology (lower numbers and lower ramification density) in mice subjected to RSD (Figure 6a,b, Figure 7a,b and Figure 9a,b). We propose that, under our conditions and the temporality of RSD, microglial cells are partially activated and have not fully reached a functional pro-inflammatory state. A more detailed analysis of morphology may resolve this paradigm. Additionally, QUIN has no effect on the area per cell in microglia across any hippocampal region. Previous studies have reported that this agonist prevents microglial activation in pro-inflammatory contexts; thus, we expected to find no effect in this group [17,32].

As observed in the GFAP results, QUIN alone reduced Iba-1 expression nearly to control levels in both the hippocampus and CTX. Further, in mice subjected to RSD, QUIN induced the same effect (Figure 6a,b, Figure 7a,b and Figure 9a,b). As expected, QUIN altered microglial morphology in response to sleep restriction. These changes were manifested as increased ramification, increased soma size, and increased cell area compared with the RSD group (Figure 6a,b, Figure 7a,b and Figure 9a,b). These findings suggest that QUIN modulates microglial activation in both the hippocampus and the cortex. These findings agree with evidence showing that D2DR exerts an anti-inflammatory effect via the Akt/GSK3β pathway, leading to microglial inactivation in the CTX [17].

In support of our findings on QUIN regulation of the inflammatory response in Iba-1 mice, D2DR-deficient mice exhibit a strong neuroinflammatory environment and increased vulnerability to MPTP toxicity. Interestingly, QUIN administration in wild-type mice prevents MPTP-induced loss of dopaminergic neurons in the substantia nigra, and this effect is modulated by the heat shock protein CRYAB [28]. Therefore, it is likely that CRYAB is responsible for the inhibition of microglia response exerted by D2DR activation by QUIN. The precise context of this molecular mechanism remains to be elucidated.

### 4.3. NF-κB Localization After RSD and QUIN Administration

The microglial response showed a consistent pattern across the CA1, CA3, DG, and CTX regions (increased Iba-1 expression following RSD and downregulation by QUIN) compared to GFAP expression. To explore the mechanism underlying microglial activation, nuclear NF-κB levels were evaluated. It has been demonstrated that microglial activation triggers the production of cytokines and other inflammatory mediators in response to different stimuli, such as LPS [20]. The production and release of these inflammatory factors are directly associated with the activation and subsequent nuclear translocation of the transcription factor NF-κB.

In this study, we demonstrate that RSD is sufficient to increase NF-κB nuclear expression in hippocampal and cortical microglial cells (Figure 10, Figure 11, Figure 12 and Figure 13). Our results are consistent with those reported by Chen and colleagues, who found a significant increase in the p-IκBα/IκBα and p-NF-κB p65/NF-κB p65 ratios in an RSD model in the CA1 region of the hippocampus [20]. Additionally, in a model of chronic sleep restriction in Sprague–Dawley rats, significant increases in phosphorylated IκBα and NF-κB p65 were observed in both the hippocampus and cortex [33]. Taken together, this evidence, along with our findings, supports the conclusion that RSD-evoked neuroinflammation is mediated by NF-κB activation.

Furthermore, studies using a chronic sleep restriction model suggest that D2DR activation by QUIN, via the CRYAB protein, also regulates neuroinflammation. Specifically, nuclear NF-κB p65 levels were reported to decrease while cytoplasmic levels increased in cells from the damaged hemisphere and hippocampus [34]. Consistent with these findings, we observed a similar effect in our model: QUIN administration decreased nuclear NF-κB expression in the mouse hippocampus and CTX (Figure 10(aIV), Figure 11(aIV), Figure 12(aIV) and Figure 13(aIV)). These results are likely mediated by a CRYAB-dependent mechanism. This chaperone targets NF-κB for proteasomal degradation in the cytoplasm, thereby preventing its nuclear translocation.

Previous results from our research group showed that systemic QUIN administration reduced proinflammatory cytokine levels (TNF-α, IL-6, and IL-1β) induced by RSD, thereby improving cognitive deficits and anhedonia in mice [18]. Based on these findings, CRYAB may represent a potential mechanism by which D2DR modulates inflammation, regulating microglial activation by inhibiting NF-κB nuclear translocation. However, the precise role of CRYAB in our model remains to be elucidated, as does its association with NF-κB activity.

## 5. Limitations and Future Directions

Although the present study shows that DRD2 activation attenuates microglial activation and NF-κB nuclear expression after REM sleep deprivation, several limitations should be acknowledged. First, the dopaminergic agonist quinpirole was administered systemically, without antagonists or specific genetic models, so it is not possible to attribute the observed effects to direct DRD2 activation in microglia. Second, this limitation is especially relevant in a REM sleep deprivation paradigm, because quinpirole-induced changes in wakefulness, stress, or sleep architecture could independently modify microglial activation and nuclear localization of NF-κB. Third, the RSD model imposes an inherent stress load, and since sleep recordings and hormonal measurements were not performed, the effect of REM sleep loss cannot be completely dissociated from the stress associated with the procedure. Furthermore, only males were studied, which limits the generalizability of the results. Finally, the involvement of CRYAB and other relevant inflammatory mediators was inferred but not directly assessed in this model, so the mechanistic interpretation remains partial and must be confirmed with selective D2/D3 antagonists, specific manipulation of DRD2 in microglia, and analysis of additional intracellular pathways.

It is worth noting that activation of D2-like receptors is linked to hypothermia in rodents [35,36]. Additionally, QUIN has been reported to decrease body temperature when administered systemically in a dose-dependent manner [36]. Despite keeping every animal at a strictly controlled room temperature, body temperature remains to be evaluated under our methodology in future experiments. Mild hypothermia has been reported to reduce cytokine release, specifically TNF-α, through decreased p38 activation and NO production [37]. Consequently, the results shown here in mice might be influenced by hypothermia. Further tests are required to establish the precise correlation between QUIN, body temperature, and sleep deprivation in our model.

## 6. Conclusions

In summary, systemic treatment with quinpirole attenuated microglial activation and reduced nuclear NF-κB expression in the hippocampus and cortex following REM sleep deprivation, a pattern consistent with the anti-inflammatory effect of D2-like dopaminergic activation in other models of brain injury. However, these findings should be interpreted as effects of systemic quinpirole treatment on REM deprivation-induced neuroinflammation, and not as a demonstration of a microglial DRD2-specific mechanism, since quinpirole alone does not allow discrimination between DRD2 and D3 contributions or between direct effects on microglia and indirect effects mediated by central dopaminergic circuits.

## Data Availability

The raw data supporting the findings of this manuscript will be provided by the authors at any time to the reviewers, and thereafter, to any researcher after publication.

## Abbreviations

The following abbreviations are used in this manuscript:

BBB	blood–brain barrier
CSF	cerebrospinal fluid
CNS	central nervous system
CTL	control
CRYAB	αB-crystallin
CTX	cerebral cortex
DRD2	dopamine D2 receptor
DA	dopamine
DG	dentate gyrus
GFAP	glial fibrillary acidic protein
IL-1β	interleukin-1β
Iba-1	ionized calcium-binding adapter molecule 1
LPS	lipopolysaccharide
NF-κB	nuclear factor kappa-light-chain-enhancer of activated B cell
NO	nitric oxide
NREM	non-rapid eye movement sleep
PBS	phosphate-buffered saline
QUIN	quinpirole
REM	rapid eye movement sleep
RSD	REM sleep deprivation
SEM	standard error of the mean
TBI	traumatic brain injury
TNF-α	tumor necrosis factor alpha
ZO-1	zona ocludens-1
